# Docosahexaenoic acid reduces sterol regulatory element binding protein-1 and fatty acid synthase expression and inhibits cell proliferation by inhibiting pAkt signaling in a human breast cancer MCF-7 cell line

**DOI:** 10.1186/s12885-017-3936-7

**Published:** 2017-12-28

**Authors:** Li-Hsuan Huang, Hsin-Yun Chung, Hui-Min Su

**Affiliations:** Institute of Physiology, College of Medicine, National Taiwan University, 1 Sec 1 Jai-Ai Rd, Taipei, 100 Taiwan

**Keywords:** Fatty acids, Docosahexaenoic acid, Arachidonic acid, Fatty acid synthase, Estrogen, Insulin, pAKT signaling, mTOR, Proliferation, Breast cancer

## Abstract

**Background:**

Fatty acid synthase (FASN), the major enzyme in de novo fatty acid synthesis, is highly expressed in breast cancer and its expression is reduced by polyunsaturated fatty acids (PUFAs) in liver. We previously found a positive association between rat mammary tumor levels of the n-6 PUFA arachidonic acid (AA) and tumor weight. We examined the roles of the major n-3 PUFA, docosahexaenoic acid (DHA, 22:6n-3), and the major n-6 PUFA, AA, in FASN expression in, and proliferation of, human breast cancer MCF-7 cells.

**Methods:**

The cells were treated for 48 h with BSA or 60 μM BSA-bound DHA, AA, or oleic acid (OA, 18:1n-9), then were incubated with or without estradiol or insulin. Western blot and ^3^H–thymidine incorporation assay were used to determine the role of DHA on FASN regulation and MCF-7 cell proliferation.

**Results:**

DHA, but neither AA nor OA, inhibits estradiol-induced and insulin-induced expression of the precursor of sterol regulatory element binding protein-1 (p-SREBP-1), its mature form (m-SREBP-1), and FASN. Estradiol or insulin stimulation increased the pAkt/Akt and pS6/S6 ratios, expression of p-SREBP-1, m-SREBP-1, and FASN, and cell proliferation, and these effects were decreased by DHA. The DHA-induced decrease in FASN expression resulted from reduced pAkt/Akt signaling and not pERK1/2/ERK1/2 signaling. In addition, DHA enhanced the inhibitory effect of LY294002 on pAkt signaling and expression of p-SREBP-1, m-SREBP-1, and FASN. However, addition of rapamycin, an inhibitor of the mTOR signaling pathways, 1 h before addition of estradiol or insulin increased the pAkt/Akt ratio and FASN expression, and this effect was inhibited by addition of DHA 48 h before rapamycin.

**Conclusion:**

We conclude that, in MCF-7 cells, DHA inhibits pAKT signaling and thus expression of p-SREBP-1, m-SREBP-1, and FASN and cell proliferation.

## Background

Fatty acid synthase (FASN; EC 2.3.1.85) catalyzes the de novo synthesis of long-chain fatty acids from acetyl-CoA and malonyl-CoA in the presence of NADPH [1]. FASN expression is undetectable in adult human tissues, but high expression is seen in hormone-sensitive tissues, including lactating breast, endometrial cell proliferation during the menstrual cycle, liver, and adipose tissue, and its expression is regulated by estrogen, progesterone, insulin, and polyunsaturated fatty acids (PUFAs) [1–3]. FASN is also highly expressed in human epithelial cancers, including breast cancer, prostate cancer, and colon cancer, in which the tumor cells, using the de novo pathway, synthesize most of their own long-chain fatty acids, which are then esterified mainly to phospholipids and are incorporated into the cell membrane for cell proliferation [2, 4–6] . FASN has been proposed as a potential drug target for chemotherapy [1, 2].

Sterol regulatory element-binding protein-1a (SREBP-1a), SREBP-1c, and SREBP-2 are members of the SREBP family of transcription factors. Unactivated SREBPs, known as precursor SREBPs (p-SREBPs), with a molecular weight of 125 kDa, are bound to the membrane of the endoplasmic reticulum and are cleaved to generate the mature active forms (m-SREBPs, 68 kDa), which translocate to the nucleus to upregulate expression of enzymes involved in the biosynthesis of fatty acids and cholesterol [2]. Expression of SREBP-1c, but not SREBP-1a or SREBP-2, is induced by insulin and stimulates FASN expression in the liver [7, 8]. In MCF-7 human breast cancer cells, SREBP-1c mRNA levels and FASN mRNA levels are high, but SREBP-1a and SREBP-2 mRNA levels are low, and, after addition of epidermal growth factor (EGF), FASN and SREBP-1c mRNA levels are increased, while SREBP-1a and SREBP-2 mRNA levels are not [9].

n-3 and n-6 PUFAs reduce SREBP-1c and FASN mRNA levels in hepatocytes [10–12]. We previously found that arachidonic acid (20:4n-6, AA) levels are 10 times higher in rat mammary tumor tissue than in the normal mammary gland, and are positively correlated with tumor weight [13]. We examined the roles of the major n-3 PUFA, docosahexaenoic acid (22:6n-3, DHA), and the major n-6 PUFA, AA, in FASN expression in, and proliferation of, MCF-7 human breast cancer cells.

Two pathways, the phosphatidylinositol-3 kinase to activate protein kinase B (PI3K/Akt) and the extracellular signal-regulated kinase 1/2 (ERK1/2) pathway, have been shown to be involved in SREBP-1c and FASN regulation [2]. One of the components of the mammalian target of rapamycin (mTOR) signaling pathways, mTOR complex 1 (mTORC1), is stimulated by insulin and plays a role in SREBP-1c regulation and cell proliferation [14]. It was therefore of interest to know which signaling pathway is involved in FASN regulation by PUFAs in breast cancer.

Most animal, cell culture, and epidemiological studies have shown that n-3 PUFAs reduce, and n-6 PUFAs increase, the risk of breast cancer [15, 16], but the mechanisms are not clear. We hypothesized that proliferation of MCF-7 cells, an estrogen-dependent human breast cancer cell, might be inhibited by DHA by reducing levels of p-SREBP-1, m-SREBP-1, and FASN and inhibiting downstream signaling, while AA would have no such effect.

## Methods

### Cell line and culture conditions

Culture media were purchased from Gibco Invitrogen (Grand Island, NY, USA), and, unless specified otherwise, all chemicals were from Sigma (St. Louis, MO, USA). MCF-7 cells were obtained from the Bioresource Collection and Research Center (BCRC number: 60,436, derived from ATCC HTB-22) (Hsing-Jue, Taiwan) and routinely cultured in Dulbecco’s modified Eagle medium (DMEM) with L-glutamine and 1 mM sodium pyruvate containing 5% fetal bovine serum (FBS), 100 U/ml of penicillin, and 100 μg/ml of streptomycin at 37 °C in a 5% CO_2_ incubator.

For experiments, the cells in DMEM containing 5% FBS were seeded in dishes or plates for 48 h, then the medium was replaced with DMEM containing 1% charcoal/dextran-stripped FBS (CD-FBS) [17] for 24 h, then with DMEM containing 5% CD-FBS or FBS supplemented with either vehicle (bovine serum albumin, BSA) or BSA-bound fatty acid [17] for 48 h, then medium, 10 nM estradiol (E_2_), or 1 μg/ml of insulin was added and the cells incubated for the indicated time. In inhibitor studies, cells were pretreated with BSA or 60 μM DHA for 48 h, then medium, 20 μM LY294002 (LY), or 0.5 nM rapamycin (Rap) was added for 1 h, followed by stimulation with 10 nM E_2_ or 1 μg/ml of insulin for 1 h or 24 h, as indicated.

### Preparation of BSA-bound fatty acids

DHA, AA and OA were solubilized by preparing a complex of the sodium salt with fatty acid-free BSA at a molar ratio of 3:1. In brief, pure DHA (7.3 μl), AA (6.6 μl), OA (6.3 μl) (Nu-Chek Prep, Elysian, MN, USA) was mixed with 0.1 ml of 0.2 M NaOH (equimolar amounts), then 0.4356 g of fatty acid-free BSA and 20 ml of 25 mM HEPES buffer, pH 7.0, were added then flushed with nitrogen and the mixture shaken for 5 h at room temperature. The BSA-bound fatty acid was filtered through a 0.22-μm filter and stored as aliquots in a − 20 °C freezer.

### Western blot analysis

The cells were scraped off and sonicated in ice in lysis buffer [20 mM HEPES, pH 7.3, containing 150 mM NaCl, 1 mM EDTA, 1% Triton X-100 and a protease inhibitor cocktail mix (Roche, USA)], then the lysate was centrifuged at 18,000 g for 15 min at 4 °C and the supernatant collected and its protein concentration measured using a Thermo Pierce BCA protein assay kit (Thermo Scientific, IL, USA). The proteins were denatured by heating at 95 °C for 5 min in SDS sample buffer, then aliquots containing 50 μg of protein were separated by 8% SDS-polyacrylamide gel electrophoresis, and electrotransferred to a polyvinylidene difluoride membrane (Bio-Rad) for 2.5 h, which was then blocked by incubation for 1 h at room temperature in 5% non-fat milk in Tris-buffered saline, 0.1% Tween 20 (TBST). The membranes were immunoblotted overnight at 4 °C with primary antibodies diluted in TBST; the antibodies used were rabbit monoclonal antibody against pAkt (1:1000), S6 (1:1000), or GAPDH (1:2000), rabbit polyclonal antibodies against Akt (1:1000), pS6 (1:1000), pERK1/2 (1:1000), or ERK1/2 (1:1000) (all from Cell Signaling), rabbit polyclonal anti-FASN antibodies (1:1000), or mouse monoclonal anti-SREBP1 antibody (1:200) (both from Santa Cruz). GAPDH was used as loading control (anti-GAPDH antibodies from Cell Signaling). Bound antibodies were detected by incubation for 1–2 h at room temperature with horseradish peroxidase-conjugated anti-rabbit (1:2500) or anti-mouse IgG (1:1000) antibodies (from Santa Cruze) diluted in TBST and an enhanced chemiluminescence Western blotting detection kit (Advansta, Menlo Park, CA, USA).

### ^3^H–thymidine incorporation assay

MCF-7 cells were seeded in 24 well plates (6 × 10^4^ cells/well) for 48 h, then were treated with BSA or BSA-bound DHA for 48 h, then stimulated with 10 nM E_2_ or 1 μg/ml of insulin for the indicated time, as described in the cell culture study. They were then washed three times with PBS and incubated with 8 uCi/ml of ^3^H–thymidine (PerkinElmer, MA, USA) in FBS-free DMEM for 3 h and the reaction stopped by addition of 10% cold trichloroacetic acid for 45 min. The ^3^H–thymidine-containing medium was removed and the cells washed three times with cold PBS, then 0.4 N NaOH was added for 1 h, when the contents of the wells were transferred to tubes containing Scintillation solution (Scintran cocktail T, BDH Chemical, Poole, UK) and counted in a beta-counter (HIDEX 300SL). All samples were tested in triplicate for one determination and the experiment was repeated 6 times.

### Statistical analysis

The data are presented as the mean ± SEM. A two-tailed Student’s test was used to compare differences between two groups, while two-way analysis of variance (ANOVA) followed by Bonferroni post hoc analysis was used to compare group effects. Statistics GraphPad Prism 5.0 (Graph Pad Software, Inc., San Diego, CA, USA) was used to perform graphical and statistical analysis. A *p* value ≤ 0.5 was considered statistically significant.

## Results

### DHA inhibits E_2_-induced and insulin-induced expression of p-SREBP-1, m-SREBP-1, and FASN in MCF-7 cells, but AA and OA has no effect

Since it has been reported that estradiol increases FASN expression in breast cancer cells and that insulin increases FASN expression in liver [2], we examined whether fatty acids could also decrease the estradiol- or insulin-induced increase in FASN expression in MCF-7 breast cancer cells.

**Fig. 1:**
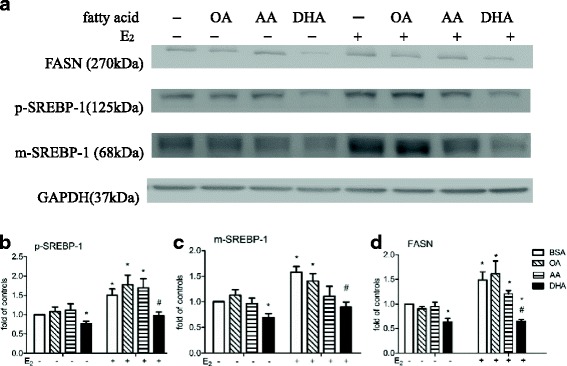
Effect of OA, AA, or DHA on p-SREBP-1, m-SREBP-1, and FASN expression in MCF-7 cells. Cells were pretreated with BSA or 60 μM BSA-bound OA, AA, or DHA for 48 h in DMEM containing 5% CD-FBS, then the same medium alone or 10 nM E2 was added and the cells incubated for 24 h, when Western blot analysis was performed to measure levels of p-SREBP-1, m-SREBP-1, and FASN (**a**). GAPDH was used as the loading control for p-SREBP-1 (**b**), m-SREBP-1 (**c**), and FASN (**d**). The levels are expressed as a fold value compared to the BSA-treated control with no E_2_ stimulation. *or # indicates a significant difference compared to cells treated with BSA without E_2_ or with E_2_ stimulation, respectively, by one-way ANOVA followed by the t-test. The data are presented as the mean ± S.E.M for 4 independent experiments

To examine the effects of OA, AA, and DHA on p-SREBP-1, m-SREBP-1, and FASN expression in untreated and E_2_-stimulated MCF-7 cells, we incubated the cells for 48 h in medium supplemented with BSA or 60 uM OA, AA, or DHA in DMEM containing 5% CD-FBS, then added the same medium alone or containing 10 nM E_2_, and incubated the cells for 24 h. As shown by Western blotting (Fig. 1), DHA, but not OA or AA, caused a significant decrease in p-SREBP-1, m-SREBP-1, and FASN expression in cells not subsequently incubated with E_2_ (left bars). Incubation of non-fatty acid-treated cells with E_2_ resulted in a significant increase in expression of all three proteins and it was rather DHA pretreatment prevented the increase in the expression of three protein, but not OA or AA (right bars). AA pretreatment caused a non-significant decrease in E_2_-induced m-SREBP-1 and FASN expression.

**Fig. 2:**
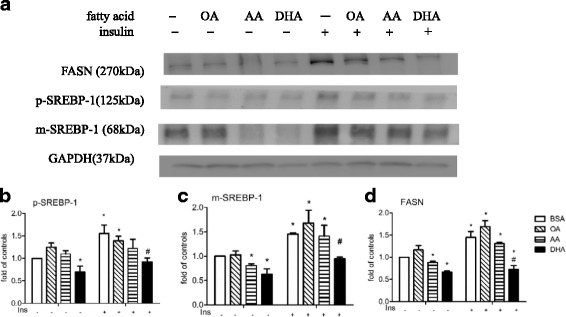
Effect of OA, AA, or DHA on p-SREBP-1, m-SREBP-1 and FASN expression in MCF-7 cells. Cells were pretreated with BSA or 60 μM BSA-bound OA, AA, or DHA for 48 h in DMEM containing 5% FBS, then the same medium alone or 1 μg/ml of insulin was added and the cells incubated for 24 h, when Western blot analysis was performed to measure levels of p-SREBP-1, m-SREBP-1, and FASN (**a**). GAPDH was used as the loading control for p-SREBP-1 (**b**), m-SREBP-1 (**c**), and FASN (**d**). The levels are expressed as a fold value compared to the BSA-treated control with no insulin stimulation. *or # indicates a significant difference compared to cells treated with BSA without or with insulin stimulation, respectively, by one-way ANOVA followed by the t-test. The data are presented as the mean ± S.E.M for 4 independent experiments

We then repeated the experiment using 1 μg/ml of insulin instead of E_2_ in DMEM containing 5% FBS, and, as shown in Fig. 2, found that, in cells not subsequently treated with insulin, DHA again caused a significant decrease in expression of all 3 proteins and AA caused a significant decrease in m-SREBP-1 and FASN expression, while OA had no effect, and that insulin again caused a significant increase in expression of all three proteins that was significantly decreased by pretreatment with DHA, but not AA or OA.

### DHA decreases the pAkt/Akt and pS6/S6 ratios, but not the pERK1/2/ERK1/2 ratio, and decreases p-SREBP-1, m-SREBP-1, and FASN expression in MCF-7 cells

**Fig. 3:**
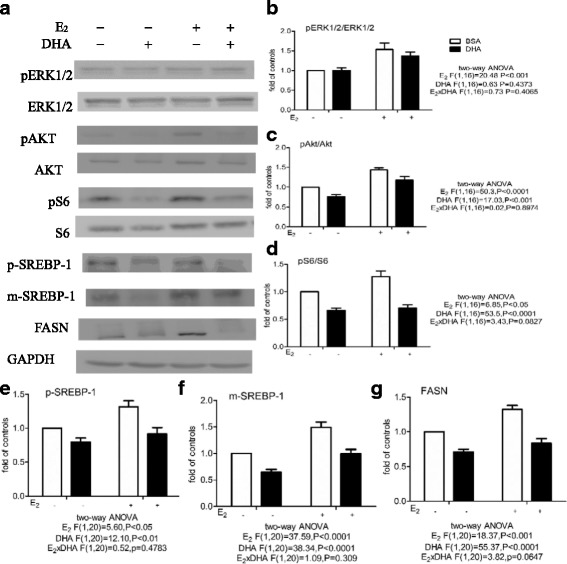
Effect of DHA or E_2_ on the pERK1/2/ERK1/2, pAkt/Akt, and pS6/S6 ratios and p-SREBP-1, m-SREBP-1, and FASN expression in MCF-7 cells. Cells were pretreated with BSA or 60 μM BSA-bound DHA for 48 h in DMEM containing 5% CD-FBS, then the same medium alone or 10 nM E_2_ was added. Western blots were then used to measure the pERK1/2/ERK1/2, pAkt/Akt, and pS6/S6 ratios after 1 h and p-SREBP-1, m-SREBP-1, and FASN expression after 24 h (**a**). Total ERK1/2, Akt, or S6 was used as the loading control for pERK1/2 (**b**), pAkt (**c**), or pS6 (**d**), respectively, while GAPDH was used as the loading control for p-SREBP-1 (**e**), m-SREBP-1 (**f**), and FASN (**g**).The levels are expressed as a fold value compared to the BSA-treated control with no E_2_ stimulation. Two-way ANOVA followed by the Bonferroni posttest was used to compare DHA and E_2_ effects. The data are presented as the mean ± S.E.M for 5–6 independent experiments

**Fig. 4:**
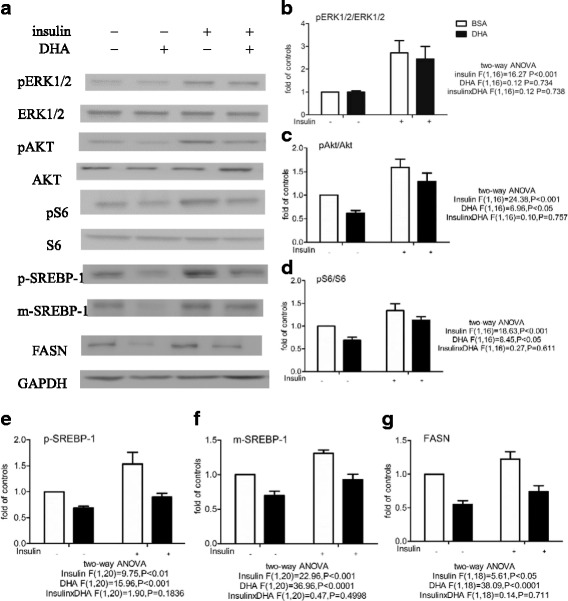
Effect of DHA or insulin on the pERK1/2/ERK1/2, pAkt/Akt, and pS6/S6 ratios and p-SREBP-1, m-SREBP-1, and FASN expression in MCF-7 cells. Cells were pretreated with BSA or 60 μM BSA-bound DHA for 48 h in DMEM containing 5% FBS, then the same medium alone or 1 μg/ml of insulin was added. Western blots were then used to measure the pERK1/2/ERK1/2, pAkt/Akt, and pS6/S6 ratios after 1 h and p-SREBP-1, m-SREBP-1, and FASN expression after 24 h (**a**). Total ERK1/2, Akt, or S6 was used as the loading control for pERK1/2 (**b**), pAkt (**c**), or pS6 (**d**), respectively, while GAPDH was used as the loading control for p-SREBP-1 (**e**), m-SREBP-1 (**f**), and FASN (**g**).The levels are expressed as a fold value compared to the BSA-treated control with no insulin stimulation. Two-way ANOVA followed by the Bonferroni posttest was used to compare DHA and insulin effects. The data are presented as the mean ± S.E.M for 5–6 independent experiments

We then examined the effect of DHA on the Akt and ERK1/2 signaling pathways without or with subsequent incubation with E_2_ (Fig. 3) or insulin (Fig. 4).

As shown by Western blotting, DHA addition had no effect on the pERK1/2/ERK1/2 ratio, but significantly decreased the pAkt/Akt and pS6/S6 ratios and p-SREBP-1, m-SREBP-1, and FASN expression. Two-way ANOVA of the results of subsequent E_2_ treatment revealed a main effect of E_2_, but not DHA, on the pERK1/2/ERK1/2 ratio, with no E_2_ x DHA interaction, and main effects of both E_2_ and DHA, with no E_2_ x DHA interaction, on the pAkt/Akt and pS6/S6 ratios and expression of p-SREBP-1, m-SREBP-1, and FASN (Fig. 3). E_2_ stimulation significantly increased all 6 values, while DHA pretreatment before addition of E_2_ had no effect on the pERK1/2/ERK1/2 ratio, but significantly decreased the pAkt/Akt and pS/S6 ratios and expression of p-SREBP-1, m-SREBP-1, and FASN. Similar results were obtained using insulin stimulation. Two-way ANOVA showed main effects of both insulin and DHA, without an insulin x DHA interaction, on the pAkt/Akt and pS6/S6 ratios and expression of p-SREBP-1, m-SREBP-1, and FASN (Fig. 4). Insulin treatment caused a significant increase in all 6 values, with DHA pretreatment significantly inhibiting the increase in the pAkt/Akt and pS6/S6 ratios and p-SREBP-1, m-SREBP-1, and FASN expression.

### DHA enhances the effect of a PI3K/AKT inhibitor or an mTOR inhibitor on FASN expression

In order to determine whether DHA could affect the inhibitory effect of a PI3K-pAkt/Akt, LY294002 or the effect of an mTOR inhibitor, rapamycin, on FASN expression and to determine the effects of these inhibitors and DHA on the factors above, cells were incubated with or without DHA for 48 h, then with or without addition of the inhibitors for 1 h, then E_2_ or insulin was added and the pAkt/Akt and pS6/S6 ratios measured after 1 h and expression of p-SREBP-1, m-SREBP-1, and FASN measured after 24 h.

**Fig. 5:**
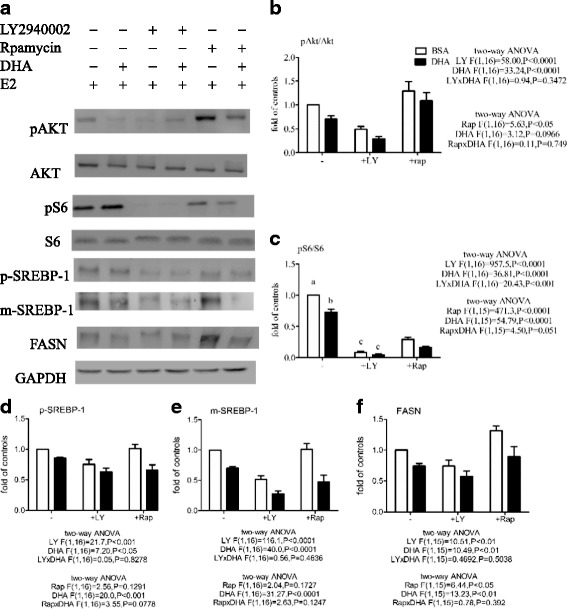
Effect of inhibitors and DHA on the E_2_-stimulated increase in the pAkt/Akt and pS6/S6 ratios and p-SREBP-1, m-SREBP-1, and FASN expression in MCF-7 cells. Cells were pretreated with BSA or 60 μM BSA-bound DHA for 48 h in DMEM containing 5% CD-FBS, then the same medium alone, 20 μM LY294002 (LY), or 0.5 nM rapamycin (Rap) was added for 1 h, followed by stimulation with 10 nM E_2_, then Western blot analysis was used to measure the pAkt/Akt and pS6/S6 ratios after 1 h of incubation and p-SREBP-1, m-SREBP-1, and FASN expression after 24 h (**a**). Total Akt or total S6 was used as the loading control for pAkt (**b**) or pS6 (**c**), respectively, while GAPDH was used as the loading control for p-SREBP-1 (**d**), m-SREBP-1 (**e**), and FASN (**f**).The levels are expressed as a fold value compared to control BSA-treated cells. Two-way ANOVA followed by the Bonferroni posttest was used to compare DHA and inhibitor effects. The data are presented as the mean ± S.E.M for 4–5 independent experiments

Using E_2_, two-way ANOVA revealed main effects of both LY294002 and DHA, without a LY294002 x DHA interaction, on the pAkt/Akt ratio and p-SREBP-1, m-SREBP-1, and FASN expression, but with a LY294002 x DHA interaction on the pS6/S6 ratio (Fig. 5). As in Figs. 3 and 4, DHA significantly decreased the pAkt/Akt and pS6/S6 ratios and p-SREBP-1, m-SREBP-1 and FASN expression. DHA enhanced the inhibitory effect of LY294002 on the E_2_-induced decrease in the pAkt/Akt ratio and p-SREBP-1, m-SREBP-1, and FASN expression, but not on the E_2_-induced decrease in the pS6/S6 ratio (the pS6/S6 ratio was reduced almost to zero by LY294002). Two-way ANOVA also revealed main effects of both rapamycin and DHA, without a rapamycin x DHA interaction, on the pS6/S6 ratio and FASN expression and a main effect of DHA, but not rapamycin, on p-SREBP-1 and m-SREBP-1 expression (Fig. 5). Rapamycin significantly reduced the pS6/S6 ratio, increased the pAkt/Akt ratio and FASN expression, and had no effect on p-SREBP-1 and m-SREBP-1 expression. DHA significantly increased the effect of rapamycin on the pS6/S6 ratio, significantly reduced the effect of rapamycin on FASN expression, and had no effect on rapamycin on the pAkt/Akt ratio.

**Fig. 6:**
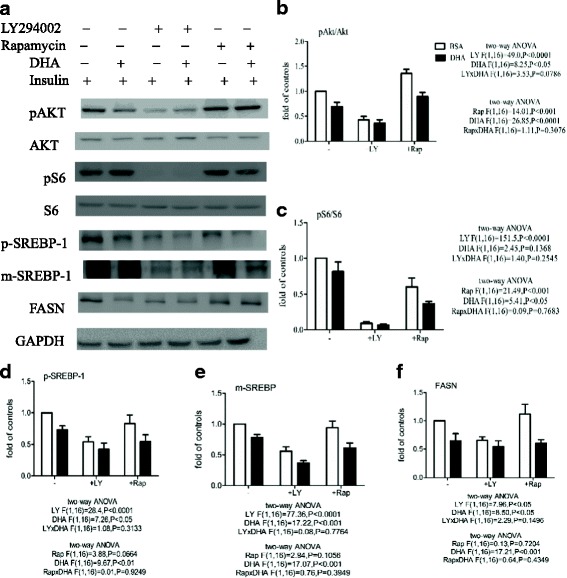
Effect of inhibitors and DHA on the insulin-stimulated increase in the pAkt/Akt and pS6/S6 ratios and p-SREBP-1, m-SREBP-1, and FASN expression in MCF-7 cells. Cells were pretreated with BSA or 60 μM BSA-bound DHA for 48 h in DMEM containing 5% FBS, then the same medium, 20 μM LY294002 (LY), or 0.5 nM rapamycin (Rap) was added for 1 h following by stimulation with 1 μg/ml of insulin, then Western blot analysis was used to measure the pAkt/Akt and pS6/S6 ratios after 1 h of incubation and p-SREBP-1, m-SREBP-1, and FASN expression after 24 h (**a**). Total Akt or S6 was used as the loading control for pAkt (**b**) or pS6 (**c**), respectively, while GAPDH was used as the loading control for p-SREBP-1 (**d**), m-SREBP-1 (**e**), and FASN (**f**).The levels are expressed as a fold value compared to control BSA-treated cells. Two-way ANOVA followed by the Bonferroni posttest was used to compare DHA and inhibitor effects. The data are presented as the mean ± S.E.M for 5 independent experiments

Using insulin, two-way ANOVA revealed main effects of both LY294002 and DHA, without a LY294002 x DHA interaction, on the pAkt/Akt ratio and p-SREBP-1, m-SREBP-1, and FASN expression, and a main effect of LY294002, but not DHA, on the pS6/S6 ratio (Fig. 6). DHA or LY294002 significantly decreased the pAkt/Akt ratio and p-SREBP-1, m-SREBP-1, and FASN expression, and DHA enhanced the effect of LY294002 on the pAkt/Akt ratio and p-SREBP-1, m-SREBP-1, and FASN expression (the pS6/S6 ratio was reduced almost to zero by LY294002). Two-way ANOVA also revealed main effects of both rapamycin and DHA, without a rapamycin x DHA interaction, on the pAkt/Akt and pS6/S6 ratios, and a main effect of DHA, but not rapamycin, on p-SREBP-1, m-SREBP-1, and FASN expression (Fig. 6). DHA decreased the pAkt/Akt and pS6/S6 ratios and p-SREBP-1, m-SREBP-1, and FASN expression, enhanced the effect of rapamycin on the pS6/S6 ratio, and inhibited the effect of rapamycin on the pAkt/Akt ratio.

### DHA decreases ^3^H–thymidine incorporation by MCF-7 cells

**Fig. 7:**
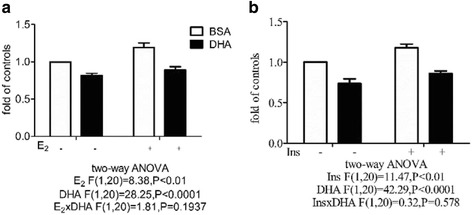
Effect of DHA on ^3^H–thymidine incorporation by MCF-7 cells. Cells were pretreated with BSA or 60 μM BSA-bound DHA for 48 h, then with or without 10 nM E_2_ (**a**) or 1 μg/ml of insulin (**b**) for 24 h, then cell proliferation were determined using the ^3^H–thymidine incorporation assay. Two-way ANOVA followed by the Bonferroni posttest was used to compare DHA and E_2_/insulin effects. The levels are expressed as a fold value compared to control cells treated with BSA with no E_2_ or insulin stimulation. The data are presented as the mean ± S.E.M for 6 independent experiments

Two-way ANOVA revealed main effects of both E_2_ and DHA, without a E_2_ x DHA interaction, on ^3^H–thymidine incorporation by MCF-7 cells (Fig. 7a) and main effects of both insulin and DHA, without an insulin x DHA interaction (Fig. 7b). DHA significantly inhibited MCF-7 cell proliferation, while E_2_ or insulin stimulation significantly increased MCF-7 proliferation, and this effect was significantly inhibited by DHA.

## Discussion

To our knowledge, this is the first study demonstrating that DHA supplementation decreases expression of p-SREBP-1, m-SREBP-1, and FASN in insulin or E_2_ stimulated MCF-7 human breast cancer cells and that AA and OA have no such effect. The increases in the pAkt/Akt and pS6/S6 ratios, expression of p-SREBP-1, m-SREBP-1, and FASN, and cell proliferation induced by E_2_ or insulin were inhibited by DHA pretreatment. The DHA-induced decrease in p-SREBP-1, m-SREBP-1, and FASN expression with or without E_2_ or insulin stimulation resulted from reduced pAkt/Akt signaling and not reduced pERK1/2/ERK1/2 signaling. In addition, DHA increased the inhibitory effect of the PI3K/Akt inhibitor Ly 294,002 on the E_2_- or insulin-induced increase in the pAkt/Akt ratio, leading to reduced SREBP1 and FASN expression. We conclude that DHA inhibits human breast cancer cell proliferation by inhibiting pAkt/Akt and pS6/S6 signaling and down regulating SREBP-1 and FASN expression.

In primary rat hepatocytes stimulated with insulin, dexamethasone, and triiodothyronine, it has been reported that, at a concentration of 250 μM, AA, linoleic acid (18:2n-6), or eicosapentaenoic acid (20:5n-3), but not OA, reduces SREBP-1c and FASN mRNA levels, while 250 μM AA, but not OA, decreases p-SREBP-1 and m-SREBP-1 expression [10] and that 500 μM AA or 20:5n-3 decreases FASN mRNA levels, with 20:5n-3 being more effective, while OA or 18:2n-6 has no effect [18]. In insulin-stimulated primary rat hepatocytes, it has been reported that 100 μM 20:5n-3 or DHA, but not OA, reduces SREBP1c and FASN mRNA levels [11], that 300 μM 18:3n-6, AA, 18:3n-3, or 20:5n-3 reduces FASN mRNA levels [19], and that 150 μM AA reduces SREBP-1 and FASN mRNA levels and p-SREBP-1 and m-SREBP-1 expression [12]. In CaCo-2 human colorectal cancer cells, 250 μM 18:2n-6, AA, 20:5n-3, or DHA, but not OA or stearic acid (18:0), reduces m-SREBP-1 protein expression and SREBP-1c_,_ SREBP-1a, and FASN mRNA levels, but has no effect on SREBP-2 mRNA and protein levels [20]. These studies show that SREBP-1 and FASN are downregulated by both n-3 and n-6 PUFAs in hepatocytes and human colorectal cancer cells. However, most of these studies focused on the effects of PUFAs on SREBP-1c and FASN mRNA levels rather than protein levels. We previously found that AA levels are 10 times higher in rat mammary tumor tissue than in the normal mammary gland and are positively correlated with tumor weight [13]. It is therefore important to distinguish the roles of DHA and AA in FASN protein expression in breast cancer. In this study, we found that 60 μM DHA, but not AA and OA, reduced p-SREBP-1, m-SREBP-1, and FASN protein expression in insulin or E_2_ stimulated human breast cancer MCF-7 cells. It is interesting to note that the anti-SREBP-1 antibody used in this study does not distinguish between SREBP-1a and SREBP-1c, but the majority of SREBP-1 mRNA in MCF-7 cells is reported to be the 1c isoform [9].

Treatment of the SKOV3 human HER2-overexpressing ovarian cancer cell line with osthole, a traditional Chinese medicine, [21] and treatment of the AU565 human HER2-overexpressing breast cancer cell line with diosgenin, a plant-derived steroid, [22], and treatment of the BT474 human breast cancer xenografts in mice with G28UCM, a FASN inhibitor [23], decreases levels of pAkt and phosphorylated mTOR, but has no effect on pERK1/2 levels, and this results in reduced FASN expression and cell viability. The PI3K/Akt inhibitor Ly 294,002, but not the ERK inhibitor PD 98059, inhibits the EGF-stimulated increase in m-SREBP-1 and FASN protein levels in MDA-MB-231 human breast cancer cells [24]. In addition, the increase in p-SREBP-1 and m-SREBP-1 protein expression and in FASN mRNA levels induced by insulin-like growth factor-1 in SEB-1 human sebocytes is blocked in the presence of PI3K/Akt inhibitor Ly 294,002, but not ERK inhibitor PD 98059 [25]. In primary rat hepatocytes, the increase in p-SREBP-1 protein and SREBP-1c mRNA levels induced by insulin is inhibited by the PI3K/Akt inhibitor wortmannin, but not the ERK inhibitor PD 98059 or the mTOR inhibitor rapamycin [26]. In LNCaP human prostate cancer hormone-independent cells, DHA reduces pAkt levels and the pS6/S6 ratio, but has no effect on the pERK/ERK ratio [27]. We found that, with or without insulin or estradiol stimulation, DHA had no effect on the pERK1/2/ERK1/2 ratio, but reduced the pAkt/Akt and pS6/S6 ratios and p-SREBP-1, m-SREBP-1, and FASN expression in, and proliferation of, MCF-7 human breast cancer cells. In addition, DHA increased the inhibitory effect of the PI3K/Akt inhibitor Ly 294,002 on pAkt signaling and p-SREB-1, m-SREB-1, and FASN levels in insulin- or estradiol-stimulated MCF-7 cells. It is suggested that the main signaling pathway involved in FASN regulation by DHA in MCF-7 cells is the PI3K/Akt pathway and that regulation of FASN expression is mainly via phosphorylation of Akt and not ERK signaling.

Rapamycin, an mTOR inhibitor, has been proposed as an anti-cancer drug [14]. In addition, a decrease in the pS6/S6 ratio, one of the major downstream targets of mTORC1, has been found to lead to increase PI3K/Akt signaling in MCF-7 and MDA-MB-468 human breast cancer cells or DU-145 human prostate cancer cells [28, 14], suggesting that the combined use of mTOR and PI3K/Akt signaling inhibitors might provide effective therapy against tumors. S6 kinase is required for the proteolytic processing of p-SREBP-1c to m-SREBP-1c, and mTORC1 increases SREBP-1c mRNA levels [29]. We found that, in MCF-7 cells, rapamycin reduced the E_2_-induced increase in the pS6/S6 ratio, but enhanced the E_2_-induced increase in the pAkt/Akt ratio and FASN levels and that these effects were inhibited by DHA pretreatment. Moreover, in both unstimulated and stimulated MCF-7 cells, we found that DHA decreased the pAkt/Akt and pS6/S6 ratios and FASN expression. DHA also decreases the pAkt/Akt and pS6/S6 ratios in LNCaP human prostate cancer cells [27]. These data suggest that DHA may be a potential drug for cancer therapy.

In studies in animals, mice fed a 20:5n-3- and DHA-rich fish oil diet, but not those fed a high carbohydrate or high OA safflower oil diet, showed a decrease in liver levels of SREBP-1c and FASN mRNAs, but not SREBP-1a and SREBP-2 mRNAs, and a decrease in p-SREBP-1, m-SREBP-1, and p-SREBP-2 protein levels, but not m-SREBP-2 protein levels [30]. Rats fed a 20:5n-3- and DHA-rich fish oil diet or an 18:2n-6-rich corn oil diet showed a decrease in liver SREBP-1c and FASN mRNA levels, whereas those fed an OA-rich olive oil or high saturated fatty acid-rich lard diet did not [10]. Finally, mice fed a 20:5n-3- and DHA-rich fish oil diet, but not those fed a 18:2n-6-rich sunflower oil diet, showed decreased liver levels of m-SREBP-1 protein [31]. Those studies show that SREBP-1 and FASN expression in the liver is downregulated in animals fed a high 20:5n-3 or DHA fish oil diet.

## Conclusions

In summary, our findings provide a potential mechanism for the decreased proliferation of MCF-7 cells caused by DHA, involving a reduction in pAkt/Akt signaling, but not p-ERK1/2/ERK1/2 signaling, and resulting in decreased expression of p-SREBP-1, m-SREBP-1, and FASN. We propose that DHA may have potential as a natural anti-breast cancer supplement that can inhibit cancer growth.

## Abbreviations

18:2n-6: Linoleic acid
18:3n-3: Linolenic acid
20:5n-3: Eicosapentaenoic acid
AA: Arachidonic acid, 20:4n-6
Akt: Protein kinase B
ANOVA: Analysis of variance
DHA: Docosahexaenoic acid, 22:6n-3
E2: Estradiol
EGF: Epidermal growth factor
ERK1/2: Extracellular signal-regulated kinase 1/2
FASN: Fatty acid synthase
LY: LY294002, PI3K/Akt inhibitor
m-SREBP: Mature sterol regulatory element-binding protein
mTOR: Mammalian target of rapamycin
OA: Oleic acid, 18:1n-9
PI3K/Akt: Phosphatidylinositol-3 kinase/protein kinase B
p-SREBP: Precursor sterol regulatory element-binding protein
PUFA: Polyunsaturated fatty acid
Rap: Rapamycin
SREBP: Sterol regulatory element-binding protein
